# Factors Influencing Mental Health of an Afghan Refugee Community in the U.S.

**DOI:** 10.1192/j.eurpsy.2025.808

**Published:** 2025-08-26

**Authors:** A. Garcia Keeme-Sayre, K. A. Chesky, A. Pathak, E. Mani, S. Essa, I. H. Hanif, S. Banu

**Affiliations:** 1 Baylor College of Medicine, Houston; 2 Harvard College, Boston; 3 Psychology, University of Houston; 4 Texas A&M; 5 Psychiatry, Baylor College of Medicine, Houston, United States

## Abstract

**Introduction:**

Rates of mental health disorders in resettled refugees surpass those of the host population (Hameed et al, KJM 2018;11 20-23). However, most studies suggest heterogenicity between populations, suggesting a need for a non-generalized approach to resettled-refugee mental health (Silove et al, World Psychiatry 2017;16 130-139). Since the Taliban assumed control in August 2021, the United States has taken in about 90,000 Afghan refugees (Green, Wilson Center 2023), 15,000 have settled in Houston, Texas (Schneider, Texas Standard 2023).

**Objectives:**

This study investigates factors that may predict higher rates of distress and symptoms of PTSD and depression within an Afghan refugee community resettled in Houston, Texas.

**Methods:**

Seventy-four Afghan refugees located in Houston, Texas were surveyed for demographic information and physical or emotional symptoms as directed by the Refugee Health Screener-15 (RHS-15). An average score of ≥ 12 on the RHS-15 or a self-reported distress score ≥5 indicated a positive result on the screening tool. Mann-Whitney and Fisher’s exact tests were used to compare differences in patient baseline characteristics and responses stratified by a positive RHS-15 or distress screen. Spearman’s Rank Correlation Coefficient was used to assess the correlation between selected response variables and outcomes as well as between the RHS-15 and distress scores.

**Results:**

40% of subjects scored ≥12 on the RHS-15 and 35% indicated a distress score ≥ 5. Income differed significantly between the RHS <12 group and the RHS ≥12 group (p=.02). The RHS <12 group had a higher proportion of individuals in the lowest income bracket, < $10,000, and a greater proportion in the $20,000-$30,000 bracket. Meanwhile, the RHS ≥12 group had a higher proportion in the $10,000-20,000 income bracket. There was a negative correlation between self-reported health scores with both RHS-15 score (ρ=-.508, p<.001) and distress score (ρ=-.423, p=.001) as well as between neighborhood support levels and distress scores(ρ=-.314, p=.018).

**Conclusions:**

This Afghan refugee cohort shows theoretical rates of PTSD and MDD higher than the average American (40.3% RHS-15 score vs. 3.6% PTSD and 8.3% MDD) (NIH 2023). The increased risk of mental disorder may be attributed to lower perceived community support, poor physical health, and low socioeconomic status. If income growth is a function of increased time spent in the United States, the unique pattern in income between the two RHS groups may reflect a “honeymoon period” that has previously been demonstrated in studies on migrants and culture shock (Maillet et al, APMH 2023; 50 563–575). Following this honeymoon period, however, lower income may predict worsened mental health in resettled refugees. These results support multi-factorial initiatives to support resettled refugees especially in areas of community interconnectedness, health and economic support.

**Disclosure of Interest:**

None Declared

